# Delayed Effects of Obese and Overweight Population Conditions on All-Cause Adult Mortality Rate in the USA

**DOI:** 10.3389/fpubh.2016.00212

**Published:** 2016-09-28

**Authors:** Albert A. Okunade, Rose M. Rubin, Adeyinka K. Okunade

**Affiliations:** ^1^Department of Economics, Fogelman College of Business and Economics, The University of Memphis, Memphis, TN, USA; ^2^Medical Center, The University of Mississippi School of Medicine, Jackson, MS, USA

**Keywords:** obesity, body mass index, overweight, all-cause mortality rate, I000, I100, I120

## Abstract

Currently, there are few studies separating the linkage of pathological obese and overweight body mass indices (BMIs) to the all-cause mortality rate in adults. Consequently, this paper, using annual *Behavioral Risk Factor Surveillance System* data of the 50 US states and the District of Columbia, estimates empirical regression models linking the US adult overweight (25 ≤ BMI < 30) and obesity (BMI ≥ 30) rates to the all-cause deaths rate. The biochemistry of multi-period cumulative adiposity (saturated fatty acid) from unexpended caloric intakes (net energy storage) provides the natural theoretical foundation for tracing unhealthy BMI to all-cause mortality. Cross-sectional and panel data regression models are separately estimated for the delayed effects of obese and overweight BMIs on the all-cause mortality rate. Controlling for the independent effects of economic, socio-demographic, and other factors on the all-cause mortality rate, our findings confirm that the estimated panel data models are more appropriate. The panel data regression results reveal that the obesity-mortality link strengthens significantly after multiple years in the condition. The faster mortality response to obesity detected here is conjectured to arise from the significantly more obese. Compared with past studies postulating a static (rather than delayed) effects, the statistically significant lagged effects of adult population BMI pathology in this study are novel and insightful. And, as expected, these lagged effects are more severe in the obese than overweight population segment. Public health policy implications of this social science study findings agree with those of the clinical sciences literature advocating timely lifestyle modification interventions (e.g., smoking cessation) to slow premature mortality linked with unhealthy BMIs.

## Introduction

Obese and overweight conditions are unhealthy for individuals and populations. Pathological body mass indices (BMIs) incubate complex sets of serious diseases, which lead to job absences and atrophied labor productivities (1), various disabilities, and all-cause mortalities in the U.S. (2) and globally. Specifically, the obesity syndrome poses major public health and policy challenges in high growth economies (e.g., China), Eastern European transition countries, and developing nations (3, 4). Moreover, obesity is comorbid with mental health conditions (5, 6), subclinical myocardial injury and heart failure (7), and other diseases. As a result, the medical personnel, public health policy makers, and global health bodies (e.g., The WHO) argue that the obesity epidemic and its premature mortality and socio-economic burdens are problematic. Interestingly, obesity is an officially certified mortality cause on 25% of the 1979–2006 death certificates in England (8). The US adult obesity prevalence rate rose from 12.7% during 1959–1962 to 29.6% in 1999–2000 (9), and remained constant for the periods 2003–2004 and 2011–2012 (10). The National Institutes of Health (11) declared overweight and obesity the second leading causes of preventable deaths following tobacco.

Obesity, an indicator of biological welfare, is defined as having a weight more than 20% above the ideal for a person. Clinically, obesity defines an excessively high amount of body fat or adiposity relative to lean muscle body mass. BMI, a frequently used but imperfect measure of obesity (12), is adult body weight in kilograms divided by height squared in meters. The currently established levels of BMI are: normal is BMI 18.5 ≤ BMI ≤ 24.9; overweight is 25 ≤ BMI ≤ 29.9; and obese is BMI ≥ 30. More than 80% of the estimated obesity-attributable deaths occur in those with a BMI greater than 30 (13). Heart diseases, cancer, and stroke, the three leading causes of US deaths [(14), p. 58], are positively correlated with significant overweight and obese BMIs. The economic and other costs of U.S. obesity are high. The direct health care cost estimates would be 25% lower when one accounts for differential mortality of the obese (15). In 1998, for example, the medical cost of obesity was estimated at $78.5 billion and the increased obesity prevalence was responsible for some $40 billion in higher medical cost through 2006, including $7 billion in Medicare prescription drug costs (16).

Currently, separate econometric model estimates of the time-phased transition of the overweight or obese conditions to mortality do not exist in the literature. Consequently, this study goal is to control for a large number of other independent effects, broaden the linkage of obesity beyond specific diseases, and econometrically isolate the robust effects of unhealthy body masses, measured by BMI levels of overweight and obese, on the all-cause mortality rate. Modeling their independent effects is crucial for their linkage to life expectancy as the all-cause mortality rate is a key measure of population health. Our study uses state-level annual data of the 50 US states and the District of Columbia (DC) to test the following statistical hypotheses. (1) Unhealthy BMIs are positively and significantly linked to the all-cause mortality rate; (2) the obese mortality impact is numerically greater than that of overweight; and (3) there are delayed effects of unhealthy BMIs on the all-cause mortality rate.

Section 2 focuses on literature review, the theoretical framework, empirical models for statistical estimation, and the data description. Section 3 covers the empirical regression results. The concluding section 4 discusses the findings and implications.

## Methods: Theory, Model, and the Data

Drawing from received theories in medicine and the allied health sciences, Lee and Skerrett (17) proposed an inverse linear dose–response link of physical activity volume (intensity, duration, frequency) to all-cause mortality rates, regardless of gender or age. More precisely, some physical activity guidelines mandating an energy expenditure of about 4200 kJU week^−1^ yields a significant 20–30% reduction in all-cause mortality risk (17). This suggests that lacking adherence to minimal physical activity guidelines, resulting in an unhealthy BMI, raises risk for all-cause mortality rate (i.e., change in mortality from a given change in obesity prevalence rate is theoretically expected to be positive). Another theory linking lifetime expenditure of energy per gram of tissue to body mass posits that tissue in smaller animals expends more energy before expiring than tissue in larger animals, and smaller individuals with higher rates of metabolism live longer than their slower and larger counterparts (18). Ervin (19) reports a U-shaped relationship between BMI and mortality, with the greatest risks in the extremes of the lowest and highest ranges.

The risk of all-cause mortality also rises with relative deprivation (20); that is, a greater sense of deprivation perceived by the poor relative to their well-off reference group leads to greater odds of ill health, disability, and all-cause mortality that are also potentially attributable to lack of quality health care access. The Gini income inequality measure is here used to proxy relative deprivation for the impoverished. Therefore, the *a priori* expected sign of the change in all-cause mortality due to wider income inequalities is positive because limited access of the poor to quality health care raises mortality risks.

Standard models of health production based on mortality rates incorporate education or schooling years as a factor (21). The better educated takes the long view in health care decision making. Therefore, the less than high school education dummy variable (*LHS*) is *a priori* expected to be associated with greater mortality (*MORT*) compared to the more educated high school or GED completion, post high school, and 4-year or higher college completion. Since income and education are highly correlated, the standard income measure is excluded from our model to avoid severe multi-collinearity. However, to capture resource constraints as a correlate of all-cause deaths, the population proportion below the US Federal Poverty Level (*FEDPVTY*) is included to test whether indigent population status (income proxy) is positively related to mortality.

Ng-Mak et al. (22) found black persons younger than 65 years to be at higher risk than others for all-cause and cardiovascular deaths. Consequently, in relation to the non-whites group, the population percent of whites (*WHITE*) is expected to be negatively associated with the all-cause mortality rate, all else equal. Moreover, all-cause mortality rate naturally tends to rise as the population ages due to general biological or soma deterioration.^1^ The aging effect of population percent ≤65 years (*POPLT65*), used here to proxy the correlation of aging structure with deaths, is expected to be negative. Moreover, the crude birth rate (*CBRATE*) is included as a distinct demographic dimension to compensate for the use of all-cause crude mortality rate, rather than standardized death rate, as the dependent variable. (One major concern when using gender- or age-adjusted death rates for the US states for multiple time periods is that standardized rates for different time periods cannot be compared because they were adjusted by different standard populations. Moreover, consistent data for each state and the DC for are unavailable.) The *a priori* sign expected of the crude birth rate in the mortality model is positive. Finally on demographics, life expectancy at birth and longevity of females tend to be higher than for males at similar age (23), for reasons, including but not limited to lifestyle risks and biological factors. Theoretically, the effect on mortality due to a change in female population proportion (*FEMALE*) is expected to be negative in our model.

Acute air pollution is a major driver of mortality (24). The environmental hazards in urban areas raise morbidity and mortality rates (25). The theoretical sign of urbanization (*URBPOP*) in the mortality model could be ambiguous, as urban areas contain both greater health hazard risks and greater concentrations and varieties of general, specialized surgical, and emergency care facilities benefiting from multiple sources of cost economies [(26), p. 83] to achieve greater efficiencies and quality of healthcare outcomes. Moreover, greater population exposure to the multiplicity of aerial gaseous, water, and solid pollutants (27) suggests that toxic waste concentrations are likely to be associated with greater all-cause mortality rates, *a priori*. Therefore, the number of solid waste sites per square mile of a US state area (*TOXIC*), exposing humans to ill health conditions, is here proposed to also raise all-cause population mortality risks.

Kromhout et al. (28) infer that a 5% reduction in saturated fat consumption, an increase of 20 mg/day consumption of vitamin C and a 10% reduction in smoking could cut the all-cause mortality rate by 12.4%. Therefore, smoking cessation and reduced tobacco use (*SMOKE*) would cut the all-cause death rate. Finally, due to some (unmeasured) permanent heterogeneous factors specific to each region, the differential effects of pathological conditions occurring in the US geographic regions are captured using regional dummies.

Our theoretical postulations together with those already established in the reviewed literature lead us to propose the following model for empirical econometric estimation:

$$\begin{array}{c} MORT_{t}=\alpha_{o}+\beta_{o}OBES_{t}+\beta_{1}OBES_{t-1}+\beta_{2}OBES_{t-2}+\beta_{3}OBES_{t-3}\\ \;+\gamma_{o}OWT_{t}+\gamma_{1}OWT_{t-1}+\gamma_{2}OWT_{t-2}+\gamma_{3}OWT_{t}{\;}_{-3}+\pi_{1}GINI_{t}\\ \;+\kappa_{1}HS+\kappa_{2}PHS+\kappa_{3}COLLG+\nu_{1}SMOKE+\lambda_{1}TOXIC\\ \;+\Lambda_{1}URBPOP+\mu FEMALE+\omega POPLT65+C_{\emptyset }BRATE\\ \;+\psi WHITE+\varphi FEDPVTY+[\text{vector of time dummies}]\tau \\ \;+[\text{vector of geographic regional controls]}\Phi +\xi \text{,} \end{array}$$

where the Greek symbols with specific regressors are the regression parameters to be estimated and ξ is the error term. The first regression model experiment has no lag effects for *OBES* and *OWT*, the second incorporates a one-period lag each for *OBES* and *OWT*, the third is a two-period lag model each for *OBES* and *OWT*, and the fourth model contains three-period lags of the pathological weight indices. The sequential lagging enables us to gain clearer insights into how increased lag lengths of unhealthy BMI ranges impact on current all-cause *MORT* rate.

The regression model was fitted to the data of 50 U.S. states and DC, taken from the *Behavioral Risk Factor Surveillance System* (BRFSS) database (29) for the 4 years 2000–2001–2002–2003 for which the BMI data are consistently reported. This study period is notably interesting from an historical perspective because, compared with the 1960–1962 period, the U.S. adult (ages 20 years and above) obesity prevalence rate rose the fastest and almost doubled in the 1988–1994 period. Coincidentally, the U.S. population diagnosed with diabetes rose the fastest and almost doubled from 8.7 million in 1995 to 8.7 million in 2005 (30). The dataset (Overview: BRFSS, 2006) is a collaborative project of the Centers for Disease Control and Prevention (CDC) and all U.S. states and territories (31). The objective of the BRFSS is to gather uniform, state-specific data on preventive health practices and risk behaviors, including weight factors, which are linked to chronic diseases, injuries, and preventable infectious diseases. The BRFSS employs telephone survey data collected from a random sample of adults by each state to measure behavioral risk factors in the adult population (age ≥18) living in households.

The dependent variable (*MORT*) is all-cause mortality per 100,000 of the adult resident population in each US state and the DC. All-cause mortality data are from the US Census Bureau (32). Separate cross-sectional annual, and panel regression models are estimated linking all-cause mortality per 100,000 of the population to “*overweight*” (25 ≤ BMI ≤ 29.9), and “*obese*” (BMI ≥ 30) adult BMI values with controls for a set of other effects (e.g., formal education, urbanization rate, density of toxic waste sites, etc.,). We estimate the distributed lag effects of obesity and overweight on the all-cause mortality in order to trace the shape of their time-phase. Separate cross-sectional regression models of the BMI effects (*OWT, OBE*S) on all-cause mortality are studied for each year, and for the panel of years. Our panel estimates would be tenable if population migration is slow across the states.

The population percent overweight (*OWT*) and obese (*OBES*) are the independent variables of core interest in this study. The other independent variables as controls, obtained from the BRFSS data, unless otherwise noted, include the *GINI* income inequality measure for each state (the Gini is an economic measure of deviation from equal income distribution with a 0–1value range); education level (*LHS* is less than high school, *HS* is high school completion or GED equivalent, *PHS* is post high school *COLLG* is 4-year college completion); and *SMOKE* is percent of cigarette smokers in each state. State specific data on crude birth rates (*CBRATE*) are from the *National Vital Statistics Reports* (33). The *TOXIC* variable is computed as the number of US Environmental Protection Agency (34) designated toxic waste sites per square mile area of each state (US Environmental Protection Agency, 2006). Finally, *URBPOP* is the percent of urban population, and the US geographic region dummies (Midwest, Northeast, and West, relative to the South) control for heterogeneous, permanent regional factors that differentially correlate with all-cause *MORT*. The base case represents normal weight, less than high school education, non-smoker, male gender, population 65 years of age and older, non-white, and the South region.

## Empirical Regression Model Results

Table 1 contains descriptive statistics of the study data. Cross-sectional data OLS regression models performed for each year had few significant coefficient estimates, high *R^2^* values of around 80% that are statistically significant (high *F* statistics) and, as expected of cross-sectional annual models, heteroskedastic errors detected using the White (35) general test (significant χ*^2^*s values at α = 0.05 level). These suggest that some distributed lag model structure would represent an improved estimation strategy, given the moderate collinearity. Moreover, while the least squares estimators are unbiased under heteroskedasticity, they are inefficient and the biased estimation of the variances invalidates statistical significance tests.

**Table 1:** **Descriptive statistics of the panel data**.

Variable	Definition	Mean	SD
MORT	Mortality per 100,000 by state	44.31373	
	Source: US Bureau of the Census, 2006		
OWT	25 ≤ Body Mass Index < 30	36.8087	1.3022
	Source: *Behavioral Risk Factor Surveillance System* (*BRFSS*), Centers for Disease Control and Prevention (CDC), 2004		
CBRATE	Crude birth rate per 1,000 population	13.8997	1.1298
	Source: *National Vital Statistics Reports* (CDC)		
OBES	Body Mass Index ≥30	21.3367	2.3897
	Source: *BRFSS* (CDC), 2004		
GINI	Gini coefficient of income inequality	0.4485	0.026
	Source: *BRFSS* (CDC), 2004		
HS	Population percent completing high school	31.9161	4.1479
	Source: *BRFSS* (CDC), 2004		
PHS	Percent of population completing post high school	27.6626	3.3533
	Source: *BRFSS* (CDC), 2004		
COLLG	Percentage of population completing 4-year college or higher	29.1729	5.4428
	Source: *BRFSS* (CDC), 2004		
SMOKE	Percent of population smoking tobacco	22.9729	3.0149
	Source: *BRFSS* (CDC), 2004		
TOXIC	Superfund toxic waste sites per square mile of the geographic land area of state	130.256	270.0375
	Source: Environmental Protection Agency, 2006		
FEMALE	Population percent female	51.0002	0.725
	Source: US Census Bureau, Population Estimates Program (PEP), 2007		
WHITE	Ethnic White population percent	17.872	13.44
	Source: US Census Bureau (PEP), 2007		
POPLT65	Population percent age 65 years and less	87.28	1.807
	Source: US Census Bureau (PEP), 2007		
FEDPVTY	Population percent below Federal poverty (PEP), 2007	12.82	3.20
	Source: US Census Bureau		
URBPOP	Population percent urbanized	71.797	14.9195
	Source: *BRFSS* (CDC), 2004		

The cross-sectional models were re-estimated using the White (35) heteroskedasticity-consistent covariance matrix estimator. The cross-sectional regression estimates suggest that a theoretically more optimal specification of the separate impacts of being obese and overweight on the all-cause mortality rate ought to account for some delayed effects of unhealthy BMIs (cumulative net energy balance for multiple time periods).

Table 2 contains the estimated generalized least squares (EGLS or cross-sectional weighted least squares) regression model results for the four model experiments. The panel data estimates for the years 2000 through 2003 across the 50 states and the DC (model I) and three variants for the subsequent years, models II (state-level data years 2001–2003), III (state-level data years 2002–2003), and IV (state-level data years 2000–2003) are presented.

**Table 2:** **Panel data EGLS regression model estimates of the determinants of US mortality rate**.

Model		I (4 years panel data, no lag)	II (4 years panel data, lagged 1 year)	III (4 years panel data, lagged 2 years)	IV (4 years panel data, lagged 3 years)

Variable Label	Independent Variable	Regression Model Parameter Estimates			
C	Constant	2.0839	18.6557	−5.7236	10.7347**
OWT_t_	Overweight in current year	2.3996^a^***	3.7804***	5.7783***	0.9619
OWT_t−1_	Overweight lagged 1 year		−0.3921	3.4297**	−0.1448
OWT_t−2_	Overweight lagged 2 years			1.0457	−1.3214
OWT_t−3_	Overweight lagged 3 years				3.7878**
OBES_t_	Obesity in current year	2.1941***	2.2607***	1.6656***	1.2056**
OBES_t−1_	Obesity lagged 1 year		0.5757**	0.3498	0.1560
OBES_t−2_	Obesity lagged 2 years			−0.4233	2.5163***
OBES_t−3_	Obesity lagged 3 years				0.6870***
CRDBRATE	Crude birth rate	0.3771	0.5368**	0.1691	0.2930***
GINI	Income inequality	2.9782^a^***	3.2623***	4.7267***	0.6220
HS	High school completion	−2.6522***	−1.9520***	0.5884	−0.1281
PHS	Post high school	−0.7805*	−0.8852**	1.0797**	−0.2003***
COLLG	4-year college degree or higher	−1.5007***	−2.0119***	−2.4399	−1.5983*
SMOKE	Cigarette smoking	0.7475	0.7598**	0.8989**	1.9810***
URBPOP	Urban population percent	2.4949***	2.6227***	2.8615***	−0.1746
TOXIC	Density of toxic waste sites	0.3037***	0.4280***	0.3201***	0.3005***
FEMALE	Female gender	1.6600	−3.5550	−4.7251	−2.3117***
POPLT65	Population age 65 and younger	−4.5355***	−6.0969***	−8.2056***	−1.4093
WHITE	White population percent	0.9116***	1.1199***	1.1788***	−0.6217
MIDWEST^b^	IL, IA, IN, KS, MI, MN, MO, NE, ND, OH, SD, WI	−0.0028	−0.0497	−0.4290*	−1.2495***
NORTHEAST	CT, DE, DC, ME, MD, MA, NH, NJ, NY, PA, RI, VT	−1.0069**	−1.0199***	−1.0962**	−1.5635***
WEST	AK, AZ, CA, CO, HI, ID, MT, NV, NM, OR, UT, WA	−0.01621	0.0523	0.1197	−0.1892***
Weighted Adjusted *R^2^*		0.9512	0.924	0.937	0.98
Weighted SE of Regression		0.6065	0.6101	0.5739	0.2703
Number of (year × state) Observations		204	153	102	51

^a^The symbols ***, **, and * signify parameter estimate statistical significance, respectively, at the 10, 5, and 1% levels.

*^b^South (comprising the states of FL, GA, NC, SC, VA, WV, AL, KY, MS, TN, AR, LA, and OK) is the base region. States within each US region are, respectively, abbreviated in column 2 of this table*.

The regression estimates in model I include current period *OWT* and *OBES* variables; model II estimation incorporates 1-period lag for variables *OBES* and *OWT*; model III includes 2-period lags, and Model IV estimates include 3-period lags. The reported model fit summary statistics are weighted. The adjusted *R^2^* values of the fitted models in this context range from 95.12% (for the no lag Model I) to 99.89% (for the 3-period lag model IV) based on panel data sets. The resulting adjusted *R^2^* of 97.14% across the models in Table 2 signals that the models in general possess several desirable attributes. First, the regression fit of the panel data models (97.905%) is far superior to those of the (misspecified and, thus, not reported) cross-sectional models (80.0%). Second, from practical statistical modeling stance, any attempt to incorporate interaction effects as additional regressors can only very marginally, if at all, raise the panel data model fit by 2.09% since that the maximum bound of the *R^2^* measure of any model fit is 1. Therefore, taken together, our estimated regression model fit to the observed panel data is quite good. Moreover, the EGLS regression estimator for this study is in general asymptotically more efficient than the classic ordinary least squares (OLS) estimator. The discussions below focus on the panel data models I (with no lags) through IV (with sequential lags) and justifies selection of model IV (with three-period lags each for *OWT* and *OBES*) as the optimal.

Current period *OWT* health state, as *a priori* expected, has a positive (models I–IV) and significant (models I–III) association with current mortality *MORT* (inverse health). Perhaps more importantly, the positive coefficient estimates of current year *OBES* across models I–IV are correctly signed and highly statistically significant throughout. In effect, the appropriately signed current *OWT* and *OBES* coefficient estimates reinforce confidence in their robustness as important correlates of all-cause deaths. Moreover, one- and three-period lagged *OWT* effects are positive and significant only in models III and IV. The mortality effects of one- to two-period lag *OBES* are even stronger: one-period lag OBES are correctly signed (models II–IV) and statistically significant (model II), two-period lag OBES are correctly signed (models II–IV) and significant (model IV), and three-period lag OBES effect is both highly significant and correctly signed (model IV). These findings suggest that a consistent history of obesity, more than overweight, tends to be associated with the current period mortality rates, *ceteris paribus*.

## Discussion

Our empirical regression results on OBES as a strong correlate of all-cause deaths is consistent with received theories in the social sciences and medicine, namely that untreated pathological BMI states gradually lead to terminal health state (mortality). This is a significant and novel finding of our study on the obesity-mortality link using panel data of the US states and the DC.

The hypothesis that reduced access of the poor to health care, captured using the *GINI* income inequality distribution proxy is positively associated with all-cause deaths is correctly signed across the models and is highly significant. This evidence agrees with Cohen et al. (36) and Kahn, et al.’s (37) that high income inequality locations are associated with high all-cause mortality risk. Theoretically, the better educated tends to articulate health information more efficiently and takes a long time horizon in decision making on health capital formation. Relative to not completing high school (base category), more schooling (*HS, PHS, COLLG*) is *a priori* expected to be inversely correlated with all-cause mortality – an hypothesis variously confirmed across panel data models (I, II and IV). Particularly, gradations of greater schooling years are associated with significantly reduced all-cause mortality rates for *HS* (models I and II), *PHS* (models I, II, and III), and for 4-year college or higher (models I, II, and IV) completion. The results are consistent with the negative association of mortality and education in previous studies (38).

Smoking, a high-risk and an avoidable lifestyle habit, is here conjectured to be positively linked with all-cause mortality. The empirical regression coefficient estimates reported in Table 2 for *SMOKE* in all the models support this assertion and they are statistically significant in models II through IV containing lagged pathological weight effects. Moreover, the ambient hazards in urban areas, *URBPOP* in general and *TOXIC* in particular perhaps also capturing lack of political voice of the poor or environmental discrimination, are positive and significant (in models I–III for *URBPOP* and in models I–IV for *TOXIC*) as theory predicts. Moreover, these estimates are broadly stable across specifications. Recall that the coefficient sign on the *URBPOP* was theoretically conjectured as indeterminate. Our empirical regression model estimates (Table 2) supports the theory that urban areas are health hazardous to humans in complex ways leading to the strongly positive correlation with all-cause deaths. This finding accords with the health economics literature in contrast to the strand of literature in industrial organization postulating that better health care facilities in urban areas raise health status.

Mortality rates tend to be associated with major demographic dimensions, such as age, gender, crude birth rate, and ethnicity. Our panel data model coefficient on ethnicity (*WHITES*) in model IV is correctly signed supports the general theory of lower all-cause mortality rate among the whites relative to other ethnicities the (base group). Consistent with the biological clinical evidence (39)^2^, the *FEMALE* gender dummy negatively associated with death rates suggests that men and women face significant differential mortality risks (40, 41). All-cause mortality is also relatively lower among the young (*POPLT65*), an effect that is uniformly correctly signed across the models (models I–IV) and significant in all but model IV. Finally, the all-cause mortality rate is highly significant and positively associated with the crude birth rate (*CBRATE*) in models II and IV.

The US regional controls (*MIDWEST, NORTHEAST, WEST*), relative to the South, suggests the presence of some highly significant permanent regional heterogeneities in all-cause mortality. These findings reinforce the standard thinking that residents of non-Southern states benefit significantly from mortality reducing permanent geographic advantages.

The empirical results in model IV (based on 2003 state-level data and incorporating current period and lag lengths of one to three periods for each of *OWT* and *OBES* along with other determinants of all-cause mortality), relative to other models (see Table 2), is optimal. Optimality of model IV is based on statistical fit (adjusted *R^2^*, etc) and consistency of the signs of the regression estimates with *a priori* theoretical expectations. Model IV results reinforce our initial conjecture that a model incorporating longer lag lengths is more appropriate for capturing the delayed effects of the BMI weight pathologies on the all-cause mortality rate.

Since income and education are typically correlated in health outcomes models, a proxy independent variable *FEDPVTY* was included as an indirect way for capturing the role of income while retaining education in the model. *FEDPVTY* surprisingly had a statistically significant negative association and so the models in Table 2 were re-estimated in its absence. Nevertheless, we present in Table A1 in Appendix (a variant of Model I) to illustrate the anomalous results with *FEDPVTY* included in Model I. Closer scrutiny suggests that the anomalous result in discussion does not derive from multi-collinearity of the *FEDPVTY* with any of the other determinants in the model. Moreover, comparing coefficient estimate of the *TOXIC* variable in Model I (Table 2), which excludes *FEDPVTY*, with that in Table A1 in Appendix, which includes *FEDPVTY*, shows the *TOXIC* variable is not picking up an omitted variable, such as poverty. In effect, the *TOXIC* parameter estimate is fairly robust (stable) across specifications (compare the numerical magnitudes and statistical significance of its estimates in Table 2 with the one reported in Table A1 in Appendix incorporating *FEDPVTY*). Despite robustness of the estimated model parameters and good fit (i.e., adjusted *R^2^* values) of the estimated models to the data in Table 2, indicating superiority relative to those in related past studies, the *TOXIC* measure may still be picking up the effect of some variable excluded from the mortality model in this study.

Several aspects of this research are innovative. First, is the use of lagged effects for the first, second, and third years for *OWT* and *OBES*, based on the time lags for the impact of weight pathology on mortality. Under certain conditions, it is possible to infer patient-level relationships from aggregate data (42). Our finding significant the lagged effects of obesity on the all-cause mortality is consistent with clinical evidence from clinical medicine that Danish patients obese in early 20s increase heart disease risk and premature mortality at age 55 (43). Second, we separate overweight and obese to study their different effects on the all-cause mortality rate. Third, we estimate cross-sectional and pooled (panel) data models incorporating the delayed pathology weight effects on mortality. In conclusion, this research augmented relevant multi-disciplinary theories and past research findings with novel insights to specify and estimate regression model parameters that link ranges of pathological BMIs (overweight, obese) to the all-cause mortality rate, for each cross-section year, and then for panel data models that included sequentially one-, two-, and three-year cumulative lags of obesity and overweight BMI as regressors. Not surprisingly, and based on the optimal model IV in this study, current and multi-period lagged obesity as well as lagged overweight conditions, among other factors, are significantly associated with the current all-cause mortality rate. The panel data model estimates yield richer insights into the delayed, compounded effect of obesity, and overweight, on the all-cause deaths. The optimal model findings are robust and many important other covariates, e.g., smoking, toxic waste exposure, demographics, schooling, and regional location, are also correctly signed and significantly correlated with the all-cause mortality rate.

Our study is based on data aggregated at the state level for the U.S. Therefore, to be consistent we focused literature review on studies based on aggregate data evidence. However, in agreement with one of the referees, it is important to also recognize related research findings from the Global Burden of Diseases (GBD) studies of individual-level data across countries. The Global BMI Mortality Collaboration (44) published recent findings on BMI and the all-cause mortality using individual-participant-data meta-analysis of 239 prospective studies on four continents (Asia, Australia and New Zealand, Europe, and North America). Their findings are that (1) all-cause mortality was minimal 20.0–25 kg/m^2^ and it increased throughout the overweight range for BMI 25.0– < 27.5 kg/m^2^ and BMI 27.5– < 30.0 kg/m^2^; and (2) for BMI >25 mortality increased approximately log-linearly with BMI. The study concludes that the broadly consistent positive association of overweight and obesity with higher all-cause mortality across continents align with various strategies instituted to arrest unhealthy BMI in many populations.

Finally, our innovative paper has some limitations for rectification in future studies. First, findings based on disease-specific mortality rates would tend to differ from those based on the all-cause mortality rates investigated here. Therefore, future studies on the differential effects of obesity and overweight population health states on premature mortality rates should focus on specific diseases^3^ (45) more closely tied to pathological BMI (such as underweight, overweight, and stages I–IV obesity ranges). Second, future mortality rate models that incorporate specific obesity control policy interventions and use longer panel datasets capturing the latest observed data points could yield better insights on public policy effectiveness. Third, future work could also incorporate the lagged effects of underweight BMI pathology as additional determinant of all-cause mortality and empirically replicate the theoretical model specification first proposed here with the use of longer data panels from other countries to test the robustness of our findings. Finally, it would be interesting to test for the possible consistency of this aggregate data research findings with the future research results from fitting the mortality rate models to less aggregated or finer data (e.g., individual, county, or regional) breakdowns.

## Key Points

- Obesity is increasingly viewed as a direct cause of mortality on official death certificates. In this paper, weight pathology in the adult population has statistically significant delayed effects that are spread over multiply years on the all-cause mortality rate.
- Controlling for a large number of potentially confounding factors, the delayed impacts of an unhealthy BMI on adult mortality rate are statistically significant for more years for the obese than the overweight.
- Our social science research findings reinforce implications from clinical evidence, that the effect of obesity on mortality can be successfully interrupted to avert premature deaths or slow the speed with which the obesity condition transits to terminal mortality.
- Our research findings reinforce the call for multipronged private–public obesity management and control strategies aimed at a timely weakening of the obesity–mortality link.

## Authors Note

A preliminary draft of this manuscript was presented in the ‘Mortality/Morbidity’ Session at the biennial meetings of the American Society of Health Economists (ASHEcon). Portions of the work of Dr. Adeyinka K. Okunade were completed at the Department of Applied Science, School of Health, Physical Education, and Recreation (HPER), Indiana University, Bloomington, IN (USA) and at The College of Medicine, University of Arkansas, Little Rock, AR (USA). The views expressed in this work do not in any way reflect those of the listed medical schools of the 3rd author. We thank Chris Ruhm, Tom Gilmer, Chad Meyerhoefer, Jeffrey Payne, and Nazmi Sari (for useful comments) and Deepraj Mukherjee (for able research assistance). Finally, we thank three anonymous referees and editors of this journal for helpful comments on earlier versions of this work. The usual caveat applies, however.

## Author Contributions

AAO and RR (health economists) initially brainstormed and came up with the research topic. AKO (Medical Doctor) was invited for his important contributions from clinical perspectives (e.g., on caloric intakes, energy balance, other clinical insights). All authors (AAO, RR, and AKO) contributed to the writing of Introduction and Literature Review merging ideas from medicine and economics perspectives. RR guided the data collection. AAO and AKO came up with the research methodology (theories). AAO performed econometric estimation and led discussions on insights. RR and AKO reviewed the computational results and all authors shared in the writing of the results and finalized the manuscript. All listed authors contributed seamlessly and consistently throughout to warrant listing of the three names as co-authors on this manuscript.

## Conflict of Interest Statement

The authors declare that the research was conducted in the absence of any commercial or financial relationships that could be construed as a potential conflict of interest.

## Footnotes

^1^Human genetics literature claims that small pieces of the telomere (compound structure at the end of a chromosome) are lost during each cycle of human DNA replication. Sequential shortening of telomere length, which can speed up through oxidative stress, is a known biomarker of ageing.

^2^The US Centers for Disease Control and Prevention found women to outlive men an average of 5 years, and a new study (40) suggests that the disparity may originate from the loss of Y chromosome in blood cells prompting men to have shorter life spans and higher cancer mortality. Interestingly, epidemiological evidence, indicating some 95% of individuals living up to 110 years are females, is further reinforcing.

^3^The range of chronic diseases associated with early deaths from physical inactivity include heart disease, stroke, type 2 diabetes, breast cancer, and colon cancer (45).

## Appendix

**Table A1:** **Panel data regression estimates of the determinants of US mortality rate: an experiment incorporating *FEDPVTY* as an additional explanatory variable in the basic model**.

Independent variable	Definition	Parameter estimate
C	Constant	63.67097***
OWT_t_	Overweight in current year	5.940647***
OWT_t−1_	Overweight lagged 1 year	
OWT_t−2_	Overweight lagged 2 years	
OWT_t−3_	Overweight lagged 3 years	
OBES_t_	Obesity in current year	1.769509***
OBES_t−1_	Obesity lagged 1 year	
OBES_t−2_	Obesity lagged 2 years	
OBES_t−3_	Obesity lagged 3 years	
GINI	Income Inequality	3.289570^a^***
HS	High school completion	–2.892221***
PHS	Post high school	–1.199448***
COLLG	4-year college degree	–2.392919***
CRDBRATE		
SMOKE	Smoking	0.248608
URBPOP	Urban population	1.943465***
COLLG	4-year college degree	–2.392919***
TOXIC	Density of toxic waste sites	0.420098***
FEMALE	Female gender	–12.62947***
POPLT65	Population age 65 and less	–4.903563**
FEDPVTY	Population below poverty	–0.685718***
WHITE	White	0.117149
MIDWEST^b^	IL, IA, IN, KS, MI, MN, MO, NE, ND, OH, SD, WI	0.221689*
NORTHEAST	CT, DE, DC, ME, MD, MA, NH, NJ, NY, PA, RI, VT	–0.933227^a^**
WEST	AK, AZ, CA, CO, HI, ID, MT, NV, NM, OR, UT, WA	–0.0232445
Weighted adjusted *R^2^*		0.94
Weighted SE of regression		0.59

*^a^The symbols ***, *, and * signify parameter estimate statistical significance respectively at the 10, 5, and 1% levels*.

*^b^South region (FL, GA, NC, SC, VA, WV, AL, KY, MS, TN, AR, LA, and OK) is base. States in the regions are, respectively, listed in table column 2*.
